# Using Machine Learning prediction to create 15 question IPV measurement tool

**DOI:** 10.1177/08862605231191187

**Published:** 2023-08-20

**Authors:** Sneha Shashidhara, Pavan Mamidi, Shardul Vaidya, Ishank Daral

**Affiliations:** Centre for Social and Behaviour Change, Ashoka University

## Abstract

Domestic violence, especially intimate partner violence, is an important issue worldwide, especially in India. Those that experience it may not always be able to come forward or have access to the required social support to act against it. We use National Family Health Survey data (n = 66,013 women) to create machine-learning models which can predict IPV instances with a recall of 78%. We use the top 15 best predicting questions that avoid sensitive issues to create a field tool that front-line health workers can use to identify women with a high risk of IPV and provide the support they need.

## Introduction

Domestic violence (DV) is a global issue where the affected suffer in silence. Recent studies suggest that as many as one out of three women globally will be subjected to some form of domestic abuse during their lifetimes (WHO, 2021). Additionally, data from a systematic review by the World Health Organization (WHO) suggests that women in South-Asian countries have a higher likelihood of suffering intimate partner violence (IPV) than their counterparts in Western countries (WHO, 2010). In India, traditional gender roles, a patriarchal society (Pew Research Center, 2022) and the extra “value” bestowed upon a male child (UNPFA, 2007) propagate a view of women as subordinates right from birth. Additionally, cultural norms such as dowry and child marriages have historically impacted women’s agency in Indian society. According to the National Family Health Survey 2015-16 (NFHS-IV), almost 30% of Indian women between the ages of 15–49 years were subjected to some form of DV in the last year. In absolute terms, this would translate into almost 100 million women, which is more than the combined populations of Australia, Canada and Spain.

In India, ML techniques have been used to build predictive models that efficiently predict women’s likelihood of experiencing IPV, either using national-level cross-sectional datasets (Mcdougal et al., 2021) or region-specific analysis (Choudhary, 2018; Ram, 2019; Rawat, 2021; Shambhavi, 2019). While these studies have established significant determinants relevant to the Indian context, there is a gap in the accurate prediction of IPV. We use nationally representative cross-sectional data of 66,013 women to create machine learning models which can predict IPV instances with 78% sensitivity. Our model comprises features that can be asked with a relatively low risk of desirability bias and operationalised by health workers in the field to identify women with a high probability of experiencing IPV.

### Literature Review

The concept of intimate partner violence has been well explored in literature, and a concise definition is provided by WHO (2012). They define IPV as *behaviour within an intimate relationship that causes physical, sexual or psychological harm, including acts of physical aggression, sexual coercion, psychological abuse and controlling behaviours. However, studying IPV proves challenging because many cases remain unreported, and victims often choose not to disclose their experiences in surveys.* Leon, Aizpurua and Rollero (2021) examined the willingness of other individuals to report IPV through vignettes in Spain and found that only 28% of respondents would report an episode of violence to the police. Additionally, a victim of IPV is perceived as less moral and more responsible for what happened than a victim of generic violence, making them vulnerable to ostracism and reputational threats (Pagliaro et al., 2021). Moreover, recent events such as the pandemic-induced lockdowns have exacerbated the issue. It is suggested that domestic violence cases in China, the United Kingdom, the United States and other countries increased after the COVID-19 pandemic began (Godin, 2020).

Studies have divided the risk factors of IPV into four levels: individual attributes (demographics, exposure to child maltreatment, mental disorder, substance abuse, acceptance of violence), relationships (multiple relationships/infidelity), community-specific (poverty, weak community sanctions) and societal (legal framework, gender and social norms). Multiple studies have found that having limited access to education is the most consistent factor associated with both the perpetration and experience of IPV and sexual violence across studies (Ackerson et al., 2008; Boy & Kulczycki, 2008; Boyle et al., 2009; Brown et al., 2006; Chan, 2009; Dalal et al., 2009; Gage, 2006; Jeyaseelan et al., 2004; Johnson & Das, 2008; Koenig et al., 2006; Martin et al., 2007; Tang & Lai, 2008). Additionally, women with more education than their husbands are more likely to be victims of IPV (Ackerson et al. et al., 2008; Xu et al., 2005). Younger women from low-income households are more likely to experience IPV (Coll et al., 2022). Separated, divorced, pregnant, or depressed women are at higher risk of victimisation. In a meta-analysis, Wang et al. (2022) showed a high prevalence of IPV among infertile women in Africa and Asia. Given the asymmetry in gender roles in India, Weitzman (2019) showed that the sex of the firstborn child is associated with domestic abuse.

Many studies have aimed to find nuanced relationships between these factors discussed in the literature and exposure to IPV. In a large-scale study in India, Boyle et al. (2009) investigated the effects of women’s education, attitudes regarding mistreatment, and living circumstances on IPV at the community and individual levels. Women’s education was found to lessen the likelihood of IPV exposure, and the link was non-linear at the individual level, with the protective influence being stronger at higher levels. Women’s attitudes toward mistreatment and living standards explained the link between women’s education and IPV at the community level. Another study (Raj et al., 2018) investigated whether women’s economic empowerment and financial inclusion predict incident IPV. The most potent effects were seen for women who had joint control over their husband’s income, with women who lost joint control being more vulnerable to IPV than those who never had control. Uthman et al. (2009) investigated the impact of socio-demographic characteristics on men’s and women’s views on intimate partner abuse against women. Under certain conditions, IPV was broadly acceptable, particularly among women, younger people, less educated, lower income, rural, with little access to the media, and single decision-makers. In addition to the studies mentioned above, another paper (Emery et al., 2017) explored the influence of spouses’ perceptions of the possibility of IPV intervention by their neighbours on the likelihood of violent perpetration through vignettes designed to induce sexual jealousy. When spouses believed there was a low likelihood of reporting IPV from their neighbour’s side, their responses suggested a higher likelihood of violence perpetration.

Machine Learning (ML) techniques have become increasingly prevalent in the prediction and identification of determinants of domestic violence (DV) as they have shown superior efficacy in uncovering risk factors and accurately forecasting instances of IPV compared to conventional logistic regression methods (Petering, 2018). Amusa et al. (2020) tried to predict factors that increase the risk of experiencing IPV using machine learning (ML) by interviewing 1816 ever-married women in South Africa. Decision Tree Learning using the classification and regression trees (CART) algorithm, Random Forest, Gradient Boosting, and Logistic regression were used for prediction. Random Forest and Decision Tree Learning outperformed, but the latter was chosen due to greater sensitivity than specificity. McDougal et al. (2021) identified new and potentially controllable elements impacting Marital Sexual Violence (MSV) in India using machine learning algorithms with qualitative thematic analysis. The NFHS-4 survey was used to obtain information regarding sexual assault experiences in marriages in the previous 12 months. The neural network model discovered factors that closely matched major themes associated with MSV found through iterative thematic analysis, including experiences of/exposure to violence, sexual behaviour, decision-making and freedom of movement, demographics, media access, health knowledge, health system interaction, partner control, economic agency, reproductive and maternal history, and health status.

Using LASSO, random forest, and boosted classification trees on a nationwide household survey data set from India, Brahma and Mukherjee (2022) found neonatal and infant mortality predictors. Given the imbalanced nature of the datasets, they use multiple techniques to balance the dataset, providing further evidence that machine learning models outperformed logistic regression in prediction accuracy for newborn death. The resampling-based approaches (RUSBoost and SMOTEBoost) yielded comparable prediction accuracies to existing interpretable ML algorithms for infant mortality. Jayachandran et al. (2021) aimed to provide a novel method for designing a short survey measure by integrating mixed data collection methods and machine learning. The study measured women’s agency through a semi-structured interview (the gold standard), a close-ended questions survey, and a lab-in-the-field test. They used LASSO and Random Forest to find the five survey questions closest to semi-structured interviews in estimating women’s agency. According to the study, random forest collected more information from five factors, potentially being the first choice of researchers. Additionally, Rituerto-González et al. (2019) used data augmentation techniques to assess the impact of stress on speech and its consequences on a speaker identification (SI) system. Their SI system achieved 96.05% accuracy using neutral and emphasised original utterances. The best results were achieved with naturally stressed samples, followed by stress-like samples created synthetically.

## Methods

### Pre-processing

We used data from the National Family Health Survey 2015-16 to build and test our models. The NFHS-4 is a nationally representative survey funded by USAID and conducted by the Indian Institute of Population Sciences and the Central Government to measure demographic and health indicators. Similar Demographic and Health Surveys (DHS) are conducted in other developing countries and are widely considered the gold standard dataset for public health researchers and demographers.

NFHS-4 was conducted with 601,509 households from all states and union territories in India. In each household, women between 15-49 years who had stayed in the house the previous night were eligible to be interviewed. In total, 699,686 eligible women were surveyed. A randomly selected subsample of 15% of households was selected to be administered the domestic violence module. One eligible woman was randomly selected to answer the module in each of these selected households. Out of these 79,729 women who completed the domestic violence module, we selected a sub-sample of women who have been in a union, totalling 66,013.

We performed a preliminary screening of the variables to ensure that the data was compatible with the algorithms and that no redundant information was passed on to the models. First, we removed all variables with more than 30% missing values. Second, we checked if there were variables in the women’s questionnaire and household questionnaire that replicated the information; wherever there was duplication, we deferred to variables from the household questionnaire. Third, we removed variables that could be interpreted as proxies for our outcome variable. This list includes the individual components of our outcome variable, whether they have been hurt by individuals other than their spouses, and instances of sexual abuse. Finally, we dropped all variables that provided meta-information about the survey, such as the enumerator information, interview day, and unique identifiers.

We were left with 652 variables for the next phase of cleaning. These 652 variables were a mix of Boolean, numeric and categorical variables. Since most ML algorithms that we used required numerical variables, we needed to pre-process these variables into forms that were acceptable to the algorithms.

Boolean variables were coded as a 0 or 1 for values False and True, respectively. We checked the distribution of the numeric variables and capped outliers at the 5 and 95 percentile levels.

Our first step in pre-processing categorical variables was to compare the distribution of the variable with our dependent variable. We then recoded to combine levels to increase the interpretability of the model. For example, multiple levels with low observations and similar values for the dependent variable were binned into a single level. We also generated a few variables encoding information about the household and respondent, for example, the household density and whether the caste of the respondent matched the caste of the head of the household.

We used self-reported exposure to physical violence as our dependent variable to build the predictive models. NFHS-4 collects information about physical violence on two levels, exposure to less severe violence and exposure to severe violence. Since the models we wanted to use work best for binary classification, we created a composite variable where exposure to either level of violence was coded as 1, and no exposure to violence was coded as 0. As a robustness check, we later compared the reported level of violence to our predicted risk probability of IPV.

### Modelling

We built and tuned predictive models using logistic regression, LASSO, random forest, and gradient-boosted decision trees.

Before beginning the fitting and hyper-tuning process, we randomly subsampled and separated 25% of the dataset as our test or hold-out set. We used the 75% dataset to train and tune our models, and the 25% test set was used to measure out-of-sample performance. An out-of-sample test set allowed us to mitigate concerns about overfitting the model and was an unbiased measure of model performance.

We demonstrate our hyper-tuning process for our random forest model below. For random forest models, the tuning parameters we iterated with were the number of estimator trees, the minimum number of samples in a node to be split on, and the maximum depth of decision trees. First, we fixed the values for two parameters: the number of features to consider at each node and a maximum number of samples to build trees. We did this by varying the values of these parameters and measuring cross-validated scores for ROC-AUC, giving us exact values for these two parameters with which we initialised the model. Cross-validation scores were highest when we used the default for the square root of the total number of features at each node and used bootstrapped samples for each tree.

Optimising the hyperparameters was an empirical question. We needed to compare the performance of models across different combinations of values for each parameter. We first ran multiple iterations with a wide range of values for each hyperparameter to narrow down each hyperparameter. We eliminated value ranges with low validation ROC_AUC scores and a large score difference for the validation and train fold. Low validation scores indicated poor predictive performance and a large difference in the validation and train fold indicated an overfitted model.

Once we had a range of values that provided good prediction without much overfitting, the grid search cross-validation technique helped compare performance for different combinations of hyperparameter values. The grid-search techniques allowed us to compare performance for a narrow range of values, providing information on model performance for granular changes in hyperparameter values. To pick the ideal model, we used the same criteria as the random search -- models with high ROC_AUC scores and low differences in train and validation scores. A list of the final hyperparameters for all the models is shown in Table S1.

Since our labels had a skewed distribution (~70/30 in favour of no domestic violence incidence), using the default threshold of 0.5 for converting class probability into class prediction was likely to underestimate the distribution of label 1 in our out-of-sample prediction. As a mitigation measure, we moved the threshold for classifying test cases as a 0 or 1 using the ROC curve. The ROC curve plotted the false positive rate (1- Specificity) versus the true positive rate (Sensitivity) for several threshold values. We used the geometric mean of Specificity and Sensitivity to measure the model’s performance for each threshold value and selected the threshold value with the highest geometric mean for Specificity and Sensitivity (Kubat, 1997; Barandela, 1993). Moving the threshold improved the out-of-sample performance of the model, from 46% recall to 72% recall.

As an additional robustness check, we created synthetic samples for the minority class and repeated the model-building exercise. We took our training dataset and ran SMOTE on it, i.e. creating additional samples of the minority class, converting the 70/30 distribution of the target class to a 50/50 distribution. We got similar performance results with a recall of 72%. We then repeated our model-building and hyper-tuning process as specified above, except for the threshold moving exercise.

## Results

We trained and evaluated multiple machine learning algorithms on the full dataset of 66013 respondents, with a 70/30 split for training and testing. Our sample was predominantly middle-aged, rural, Hindu, lower-caste and lower-income groups. The median age of respondents and spouses was 32 and 36, respectively. 71% resided in rural areas, and 75% of the sample was Hindu. 79% of the sample were part of Other Backward Caste, Scheduled Caste or Scheduled Tribe categories. 52% of the sample had completed secondary school, and 33% had worked in the last 12 months.

Given the ubiquity, ease of interpretability, and straightforward execution of logistic regression, we used its performance as the baseline to benchmark other algorithms’ performance. The positive recall rate in our test sample for a logistic regression model was 63%. We built on the logistic regression by introducing two regularisation parameters, L1 type and L2 type. L1 type regularisation, more commonly known as LASSO regularisation, performed significantly better, with a positive recall rate of 74%. L2 type regularisation, on the other hand, matched logistic regression’s performance of 63%.

Next, we trained models on tree-based algorithms, with Random forest models performing at 72% and gradient-boosted trees giving a relatively high performance of 77%.

**Figure 1.**
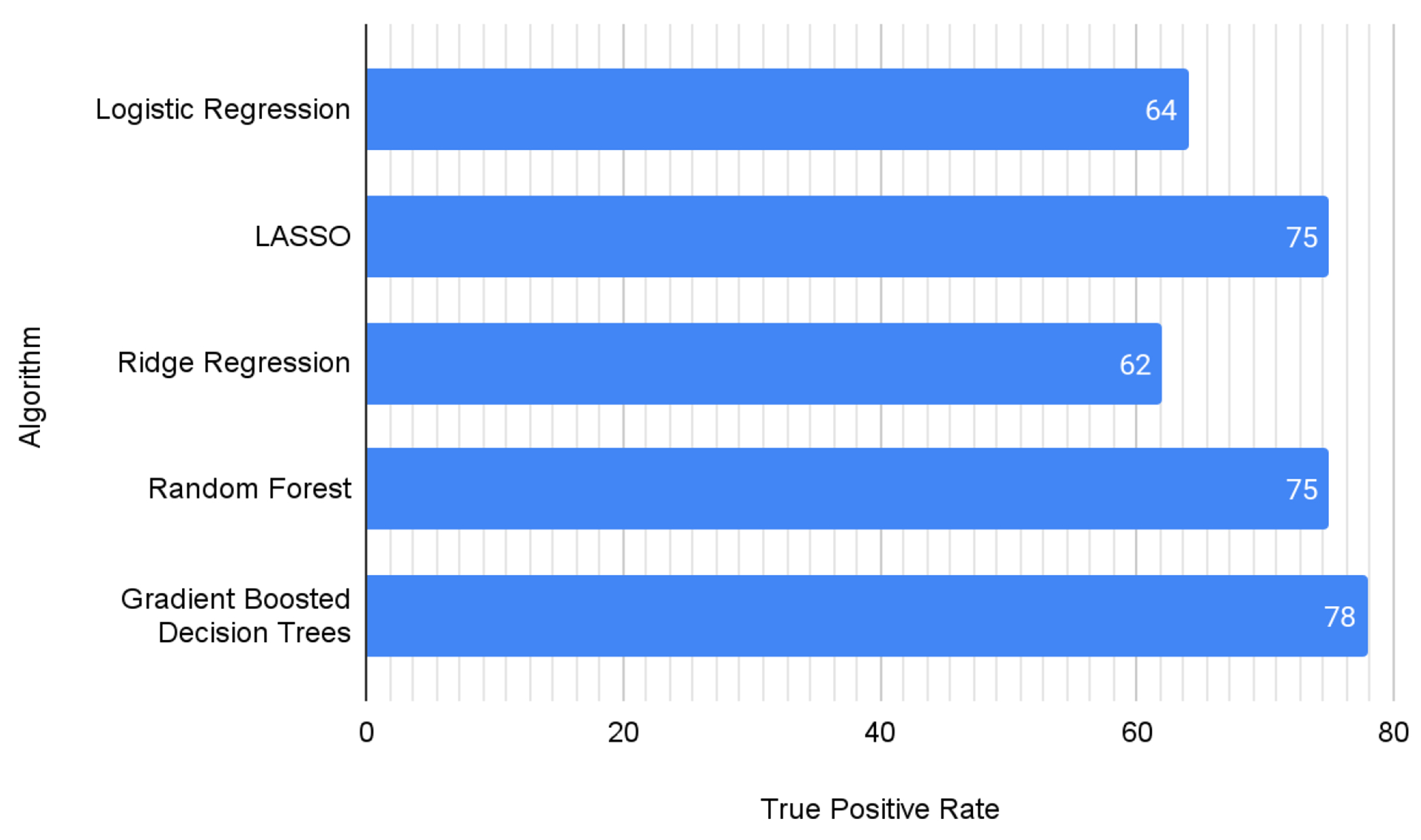
True Positive Rate for each of the different algorithms Note: This figure shows the positive recall rate for all the algorithms tested, and it can be observed that LASSO, random forest, and gradient-boosted tree algorithms have the best performance

Figure 1 shows the positive recall rate for all the algorithms tested, with LASSO, random forest, and gradient-boosted tree algorithms providing some of the best performance. Table 1 describes all precision and recall metrics for each label class, the model’s weighted precision and recall scores, and the weighted F1 score. The F1 score serves as a balanced measure of model performance, and we calculate the weighted average of the F1 score, where the ‘weight’ used is the proportion of each class’s occurrence. The same three models of LASSO, random forest and gradient boosted tree score the highest in all the metrics.

Table 2 shows the top 30 predictors for Random Forest Model. Figure 3 compares feature importance across our three best models to understand the major thematic predictors of domestic violence. Objective variables that describe the husband’s and father’s behaviour and the woman’s circumstance are some of the best predictors of IPV. These include the two most significant predictors of IPV for married women: alcohol consumption by the husband and prior exposure to intergenerational violence (e.g. if the woman’s father ever hit her mother). Other variables in this category include marital separation and miscarriage. The wife’s perception of the husband’s control issues, such as the husband being jealous of the wife talking to other men, him insisting on knowing her location, him accusing her of being unfaithful, and limiting her family visits are some of the best predictors across the three best models.

Interestingly, both women saying they are never afraid of their husbands and saying they are afraid most of the time are predictors of IPV. Another important category of variables is women justify IPV for the wife showing disrespect or arguing and neglecting children. These are part of the top 15 predictors for all three models.

Demographic descriptors such as the relative household wealth, measured as a composite of the consumer durable goods the household possesses, is a significant predictor, as well as the household having a mattress and a refrigerator. In addition to household-level demographic descriptors, individual-level predictors such as education level and ability to read a whole sentence for the respondent are important predictors of IPV, with lower socioeconomic levels predicting a higher incidence of IPV. Temperature measures significantly predict high IPV locations among the cluster-level geospatial covariates.

We repeated this exercise for the random forest model by removing all the questions in the DV module and those that could be considered ‘sensitive’, i.e., answers that the respondent might not be comfortable sharing and therefore give socially desirable answers, especially when they do not match the actual answers. Table 3 provides a list of questions/features omitted in this set. The top predictors are demographic and cluster-level factors. As seen before, the woman’s education, ability to read a whole sentence, and household wealth are factors. Besides, the husband’s education and household-level smoking indicators and owning a pressure cooker are top predictors. Different temperature indicators are also significant, with higher mean temperatures predicting IPV.

**Figure 2:**
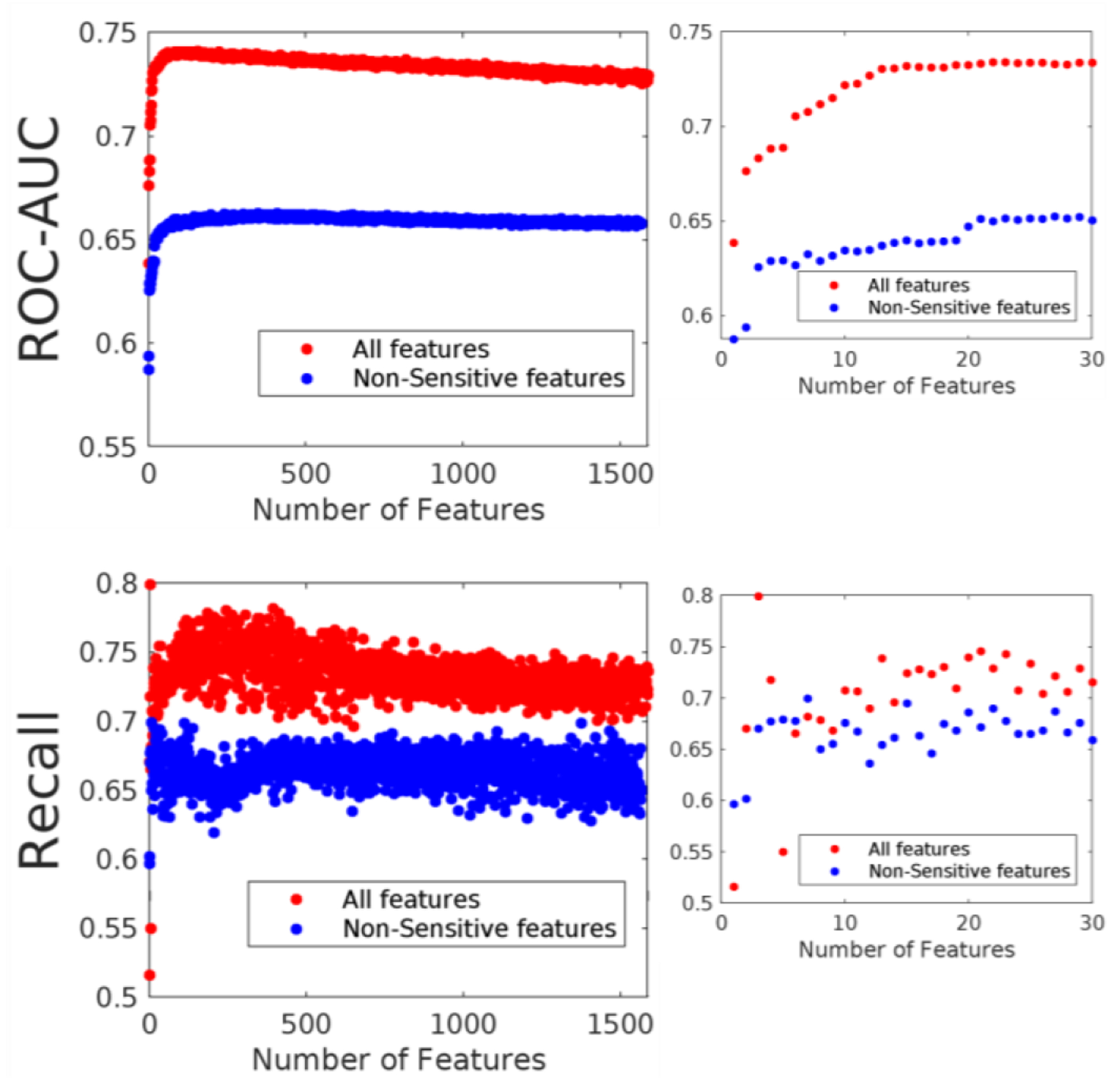
Performance of random forest models based on the number of features included Note: The top row of the figure denotes the ROC-AUC progression with an increasing number of features inputted in random forest, and the bottom row denotes the recall progression with an increasing number of features inputted in random forest

**Figure 3:**
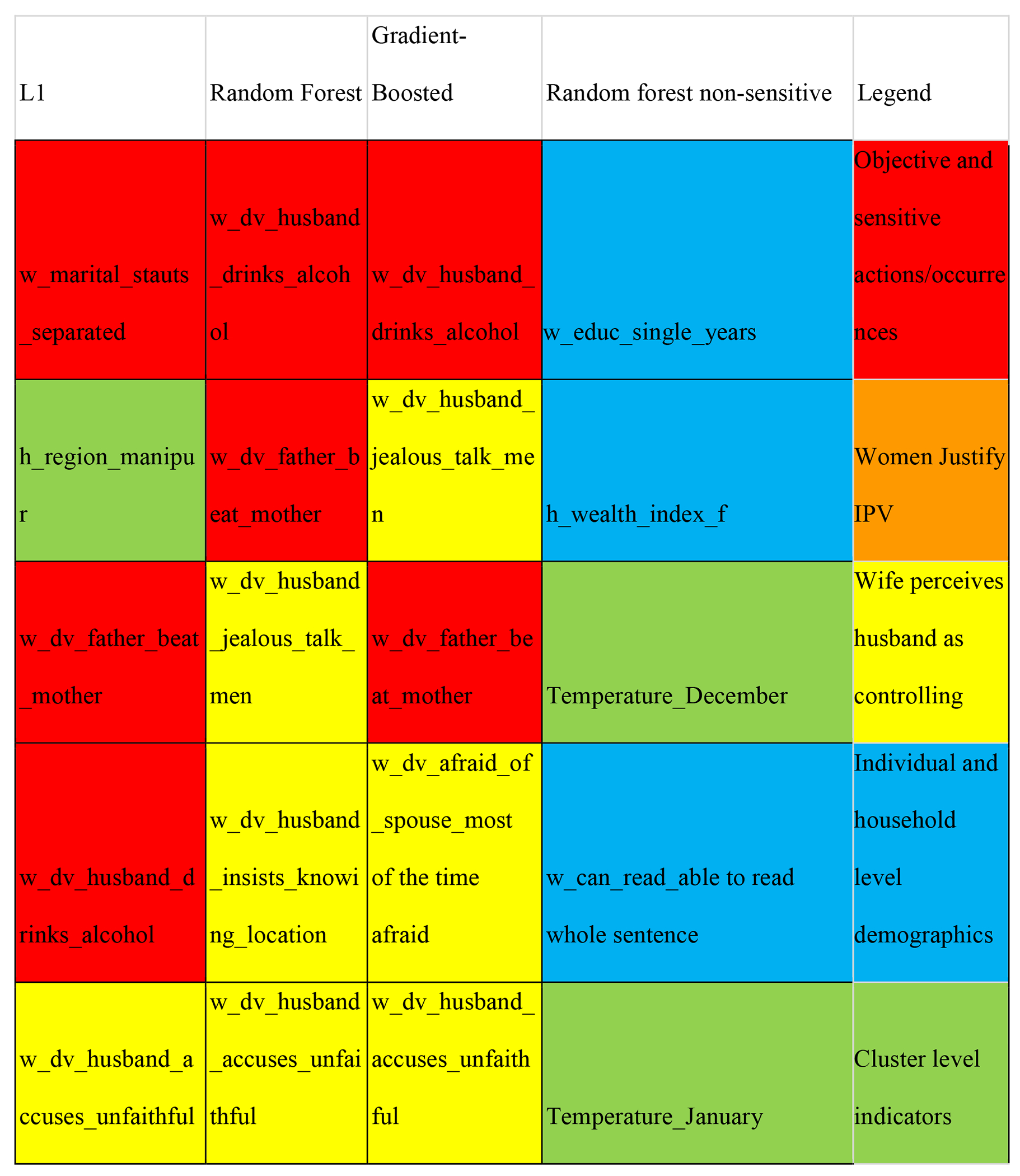

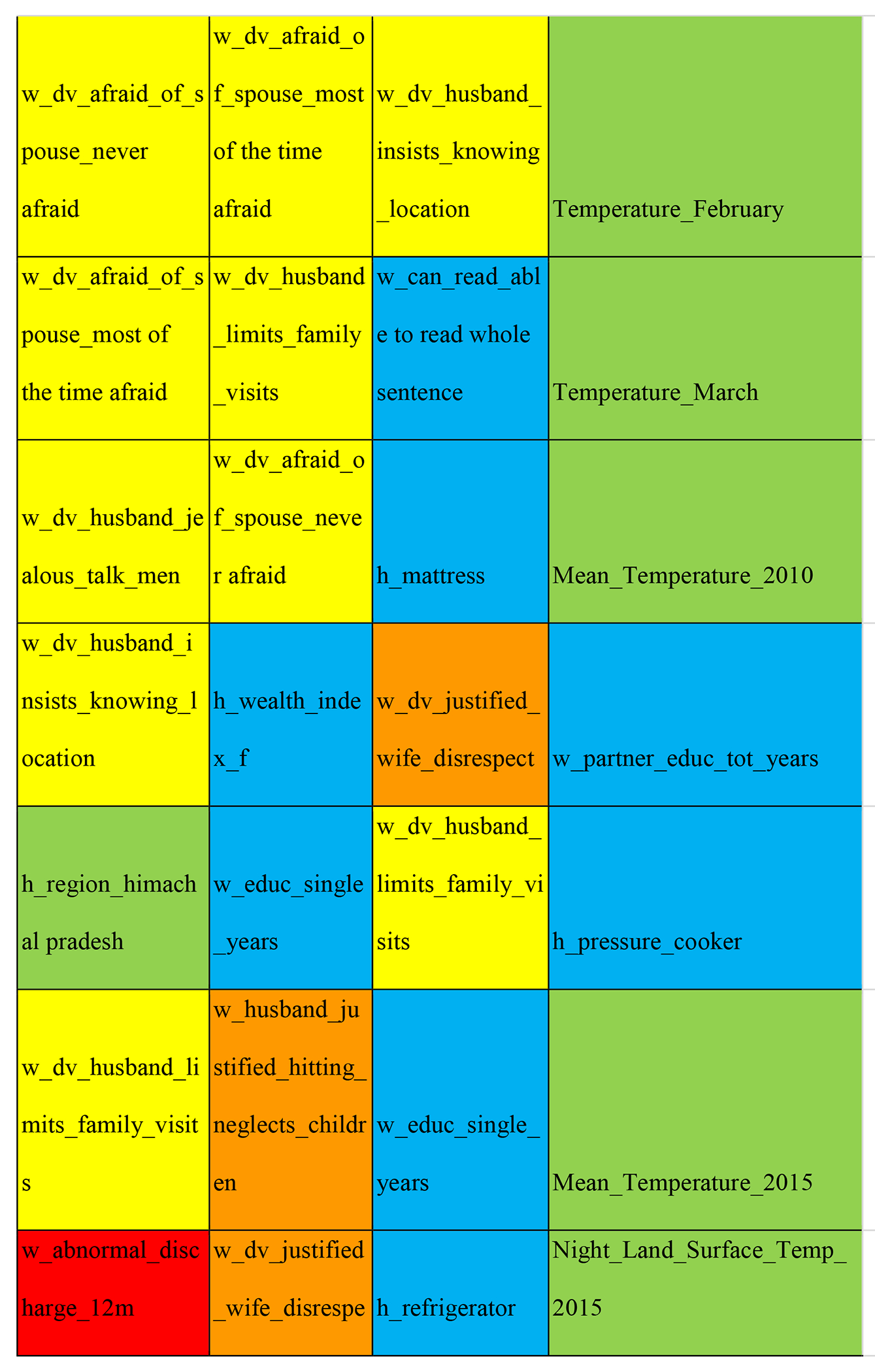

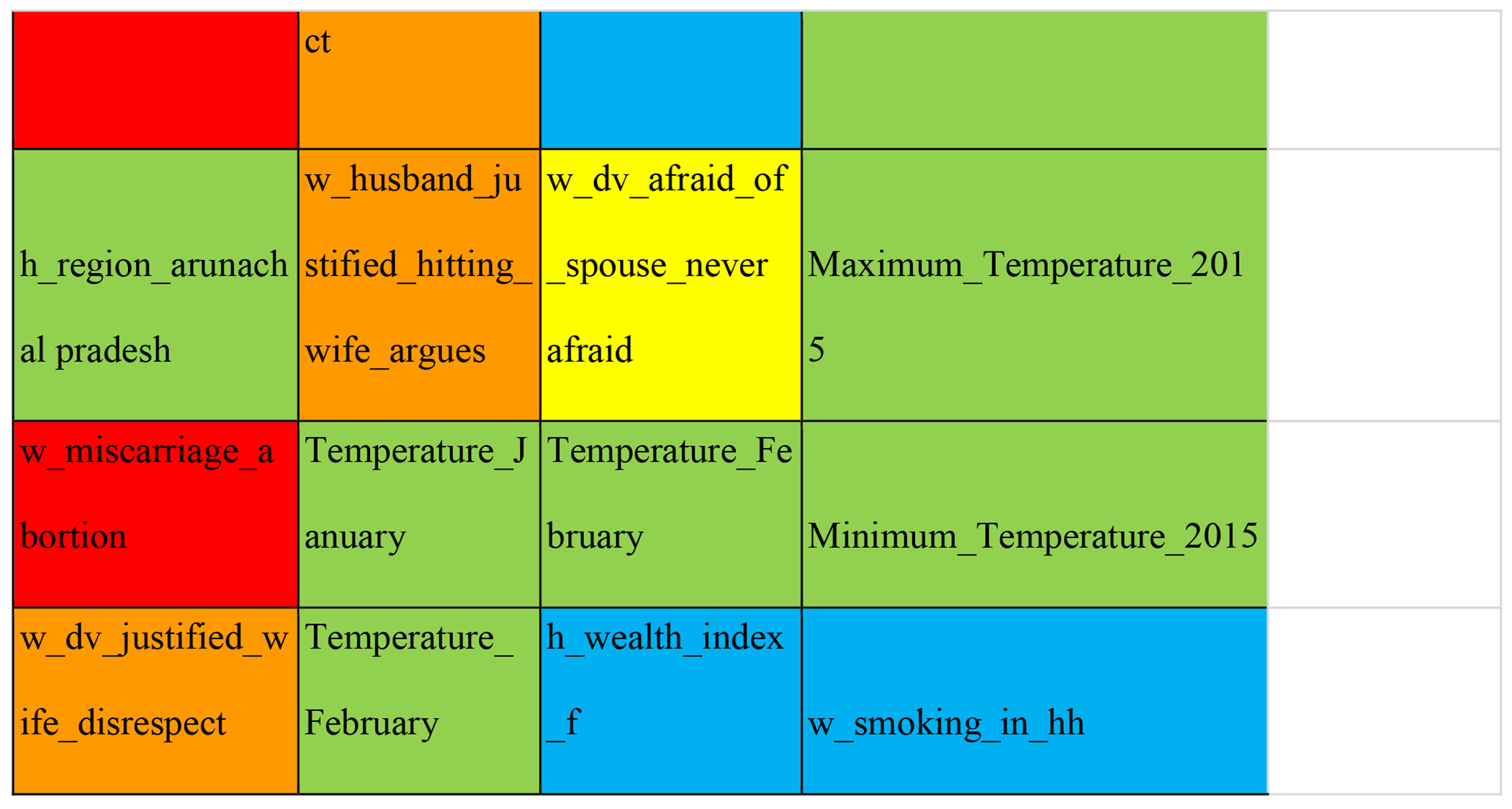
Comparison of the top 15 features across the best-performing three models. Note: This figure highlights the top 15 features chosen by all the models. It helps understand the significant predictors of domestic violence thematically with the colour red denoting “sensitive questions”, the colour orange denoting “instances of women justifying interpersonal violence, the colour yellow denoting wife’s perception of the husband as controlling, the colour blue denoting individual and household demographic questions and the colour green denoting cluster level indicators.

Next, we aimed to find the top 30 questions with the best predictive performance. We ran iterative models, adding features in sequential order of importance from the random forest model and observing how the performance changes depending on the number of features selected (Figure 2). This process was done with and without sensitive features. We see that performance in terms of recall rate and ROC-AUC increases as more features are added in the model, both in the case of all features and when using only non-sensitive features, up to a limit of about 50 features, where performance starts plateauing out. There is a noticeable decrease in performance of 8–10% when sensitive features are omitted, which persists even when 300 features are used.

Lastly, we tested the robustness of our model by understanding the nature of false positives and negatives. The NFHS-4 survey’s domestic violence module questions allow us to classify women as not experiencing IPV, experiencing low- severity, and experiencing high severity. The low and high severity were clubbed as the IPV class for model training purposes. Table 4 shows the confusion matrix for the random forest. We see that very few of the high-severity cases are classified as NO IPV (14.7%). The probability the model assigns is greater for the high-severity cases, even though the model has not directly trained on the difference between high and low-severity cases.

## Discussion

Building machine learning models provides one way to systematically assess the relative importance of predictors from a high-dimensional feature space. In this study, we attempt to isolate the best predictors of intimate partner violence among married couples across India. We train our model on a representative dataset – the National Family and Health Survey 2016-16, providing what we think are reasonably generalisable insights into IPV in India. The two most important predictors of IPV are alcohol consumption by the husband and exposure to intergenerational violence by the wife. Alcohol use has been well known to be a predictor of domestic violence (Hagelstam & Häkkänen, 2006; Mayshak et al., 2020), hypothesised to be related to IPV in multiple ways (Sontate et al., 2021). It lowers cognitive function and self-control and causes failure to resolve conflicts in nonviolent ways (Beck et al., 2013). It is a confound in that excessive drinking causes poor decision-making, exacerbating financial situations (Greene et al., 2017).

Women who think spousal violence inflicted by husbands is justified in certain perceived transgressions, such as if the wife neglects her children, argues with the husband, burns food or goes out of the household, are associated with IPV. Prior literature on the directionality of such self-blame amongst IPV victims is mixed. Psychology literature suggests that self-blame in IPV victims is a consequence of violence. It is associated with low self-esteem and post-traumatic stress disorder among IPV victims and victims of sexual abuse (Beck, 2013; DeJonghe et al., 2008). Reich 2014 suggests that self-blame in IPV victims is a coping strategy for violence. Believing that the woman’s transgressions lead to deserved punitive actions from the spouse makes these actions wholly preventable, thus providing victims with a superficial level of control over the violence. Another view on self-blaming states that exposure to intergenerational violence in the formative years for the wife and spouse could reinforce patriarchal social norms of wives being of low status in marriage (Choi, 2020).

In addition, multiple measures of controlling behaviour by the husband in the relationship are strong predictors of violence, including the husband not trusting the wife with money, insisting on always knowing her location and not allowing the wife to meet family or friends. Such behaviour can be linked to a desire by the husband to reduce the wife’s autonomy and mitigate any exit options the wife may have from the relationship. Controlling behaviour can also be seen as a reaction to the perceived threat of infidelity, which our models corroborate – women whose husbands accused them of being unfaithful or were angry if they talked to other men were at higher risk of domestic violence (Kyegombe et al., 2022). Among demographic indicators, lower levels of wealth and education are associated with IPV, and cluster-level indicators of high mean temperatures are also predictive. A study in Spain also showed an increased risk of IPV with heat waves. They found that, independently of the trend and seasonalities, a positive and statistically significant relationship existed between the different IPV indicators analysed and the maximum temperature reached during heat waves (Sanz-Barbero et al., 2018).

Using the random forest algorithm, we create a list of 15 questions that predict IPV with 74% recall (Figure 2). It is essential because it may not be possible to replicate the exhaustive nature of data collection as the NFHS survey questionnaire in the field. We then omit questions that could be construed as sensitive (Table 3) and thus lead to less honest answers from respondents and generate a set of 15 non-sensitive questions that predicts IPV with 63% recall. While there is a sizable difference in predictive power between the two sets of questions, using the set without sensitive questions might give us more reliable data.

While fitting machine learning models, there is always the danger of overfitting models on the training dataset, leading to poor out-of-sample performance. To mitigate this error, we train our models using cross-validation methods over five splits of the train data set. We compare the ROC-AUC score between the four train splits and one validation split to ensure our hyper-parameter search does not provide overfitted models. Since our target variable is naturally skewed towards value 0 (~ 70:30), we use synthetic minority oversampling to create a balanced dataset as a robustness check for our model. Training on these ‘balanced’ datasets provides similar out-of-sample performance (68%), where the test split skews towards value 0.

We perform additional robustness checks to ensure our final model provides reliable estimates. First, we generate a set of plausible responses to the feature set by randomly selecting the value of each feature from the complementary superset of values taken by the particular feature for each row for the test split. Predicting the domestic violence incidence for this synthetic dummy dataset gives us inferior performance (39% recall for a positive class using random forest), suggesting that our prior performance was meaningful and based on the pattern of responses. Secondly, we check for performance when all features take random values for each row. This randomly generated test split feature set shows an overwhelming bias to classify respondents as victims of domestic violence (94% of the test set is classified as a positive label class), again indicating the model picking up on an actual pattern. We use measures such as F1 scores as it balances precision and recall and minimises false positives and false negatives. Achieving a high F1 score would imply a good trade-off between identifying all actual high-risk cases and avoiding false alarms, as achieved by LASSO and the gradient-boosted decision trees. Lastly, the model is less likely to wrongly classify a high-severity IPV case as no-IPV, suggesting the algorithm is based on authentic patterns underlying IPV.

Although our study provides valuable insights into the predictors of intimate partner violence (IPV), it is crucial to consider contrary findings and potential fallacies. The study identifies alcohol consumption by the husband and exposure to intergenerational violence by the wife as the most significant predictors of IPV. However, it is necessary to acknowledge that there are divergent views on the relationship between alcohol use and IPV, with some studies suggesting a causal link and others highlighting complex interdependencies (Kearns et al., 2015). Controlling behaviour by the husband and accusations of infidelity are identified as strong predictors of IPV, yet it is crucial to consider the potential biases and limitations inherent in self-reported data. One of our models’ major limitations is that the label/ground truth is based on self-reports (Follingstad, D., 2017). Our models are based on only those cases of IPV where the woman can also self-report IPV. Cases where the women were not forthcoming but did experience IPV are labelled ‘no-IPV’ in the dataset. Thus, our models might pick up characteristics of women that can come forward about their experience rather than characteristics common to all women enduring IPV. To this end, we propose to validate further our models on data where the incidence of IPV is established through independent means. We could implement a format where the women answer these questions anonymously, reducing bias (Sanmani L et al., 2013). Another possibility is to approach others in the women’s social network to ascertain the incidence of IPV (Latta & Goodman, 2011; Parker, 2015). Further, we could use Keynesian beauty contest like methods to incentivise truth from the participants.

We further plan to test both sets of questions, with and without sensitive features, to empirically answer the question of which set fares better in a real-world scenario, the one with greater predictive power or the one where the data is potentially more reliable.

Our results validate the important thematic areas in the study of IPV through a large-scale representative survey in India. While these areas have been investigated before, combining them into one study allows for a prioritisation exercise to isolate the most critical predictors of IPV and the areas to develop interventions against IPV. Our analysis opens at least two major avenues for further investigation and interest from researchers and policymakers. Firstly, self-blame by IPV victims and exposure to inter-generational violence are understudied predictors of IPV. More research is required to understand the direction of causality between these predictors and IPV incidence. While causal linkages may be challenging to investigate immediately, a first step could be understanding if accepting interpersonal violence decreases women’s agency. Secondly, it is well understood that domestic violence is severely underreported through formal channels. Women fear social stigma, humiliation, familial discord, and even physical backlash due to reporting domestic violence. In such a scenario, predicting the quantitative risk of IPV based on responses to less-sensitive questions could help provide women with plausible deniability and reduce informational asymmetry between institutional support and IPV victims. Our model performs well with a limited set of reasonably non-intrusive features and could potentially be tested in the field as a diagnostic tool.

## Figures and Tables

**Table 1:** Key Performance Metrics for each of the algorithms used

Model	Precision == 1	Recall == 1	Precision == 0	Recall == 0	Weighted Precision	Weighted Recall	Weighted F1 Score
Logistic Regression	38	64	81	58	69	60	62
LASSO	53	75	88	74	78	74	75
Ridge Regression	38	62	80	60	68	61	63
Random Forest	49	75	88	70	77	71	72
Gradient Boosted Decision Trees	52	78	89	71	79	73	75

Note: This table describes all precision and recall metrics for each label class (presence or absence of domestic violence), the model’s weighted precision and recall scores, and the weighted F1 score where the ‘weight’ used is the proportion of each class’s occurrence.

**Table 2:** Top 30 Features for Random Forest Model

Sr. No.	Variable	Absolute Importance Score	Relative Importance Score
1	w_dv_husband_drinks_alcohol	0.06761791074	100
2	w_dv_father_beat_mother	0.06154565808	91
3	w_dv_husband_jealous_talk_men	0.05638685066	83.4
4	w_dv_husband_insists_knowing_location	0.03656452309	54.1
5	w_dv_husband_accuses_unfaithful	0.03557638138	52.6
6	w_dv_afraid_of_spouse_most of the time afraid	0.03219068917	47.6
7	w_dv_husband_limits_family_visits	0.02421397745	35.8
8	w_dv_afraid_of_spouse_never afraid	0.0197027401	29.1
9	h_wealth_index_f	0.01568631366	23.2
10	w_educ_single_years	0.01440923212	21.3
11	w_husband_justified_hitting_neglects_children	0.01320150962	19.5
12	w_dv_justified_wife_disrespect	0.0126069734	18.6
13	w_husband_justified_hitting_wife_argues	0.01148511319	17
14	Temperature_January	0.01132130908	16.7
15	Temperature_February	0.01094270905	16.2
16	Temperature_December	0.01036571703	15.3
17	Temperature_November	0.009728378301	14.4
18	w_partner_educ_tot_years	0.009431869176	13.9
19	Temperature_March	0.008630751266	12.8
20	Mean_Temperature_2010	0.008436909441	12.5
21	w_can_read_able to read whole sentence	0.008010589123	11.8
22	Mean_Temperature_2015	0.007960940777	11.8
23	h_pressure_cooker	0.007943338995	11.7
24	w_dv_husband_distrust_money	0.007372121689	10.9
25	w_dv_husband_limits_meeting_friends	0.007267339058	10.7
26	h_share_with_other_hh_missing	0.006574977155	9.7
27	w_husband_justified_hitting_wife_goes_out	0.006567287998	9.7
28	h_cluster_altitude	0.006309642255	9.3
29	Minimum_Temperature_2015	0.006097748328	9
30	Night_Land_Surface_Temp_2015	0.005959677072	8.8

Note: This table describes the top 30 features selected by the Random Forest Model and their respective importance scores

**Table 3:** Excluded Predictors of Sensitive Nature

Sr No	Feature
1	w_dv_husband_drinks_alcohol
2	w_dv_father_beat_mother
3	w_dv_husband_jealous_talk_men
4	w_dv_husband_accuses_unfaithful
5	w_dv_husband_insists_knowing_location
6	w_dv_afraid_of_spouse_most of the time afraid
7	w_dv_husband_limits_family_visits
8	w_dv_afraid_of_spouse_never afraid
9	w_dv_afraid_of_spouse_sometimes afraid
10	w_dv_justified_wife_unfaithful
11	w_dv_justified_wife_disrespect
12	w_husband_justified_hitting_neglects_children
13	w_husband_justified_hitting_wife_argues
14	w_dv_husband_limits_meeting_friends
15	w_dv_husband_distrust_money
16	w_husband_justified_hitting_wife_goes_out
17	w_husband_justified_hitting_wife_burns_food
18	w_age_first_sex_imputed
19	w_age_at_first_intercourse_cleaned

Note: This table describes the list of questions of sensitive nature which were omitted to check the model’s performance without them.

**Table 4:** Random Forest predictions across the severity of cases

		Predicted	
		no	yes
actual	no	78.2%	21.8%
	medium	35.3%	64.7%
	high	14.7%	85.3%

Note: This table shows the confusion matrix for the random forest model. It can be observed that only a few of the high-severity cases (14.7%) are categorised as no Interpersonal violence.
